# Polymeric Drug Delivery Systems Bearing Cholesterol Moieties: A Review

**DOI:** 10.3390/polym12112620

**Published:** 2020-11-06

**Authors:** Paweł Misiak, Karolina H. Markiewicz, Dawid Szymczuk, Agnieszka Z. Wilczewska

**Affiliations:** Faculty of Chemistry, University of Bialystok, Ciolkowskiego 1k, 15-245 Bialystok, Poland; k.markiewicz@uwb.edu.pl (K.H.M.); d.szymczuk@uwb.edu.pl (D.S.)

**Keywords:** cholesterol, polymers comprising cholesteryl moiety, drug delivery, drug encapsulation, encapsulation efficiency, encapsulation capacity

## Abstract

This review aims to provide an overview of polymers comprising cholesterol moiety/ies designed to be used in drug delivery. Over the last two decades, there have been many papers published in this field, which are summarized in this review. The primary focus of this article is on the methods of synthesis of polymers bearing cholesterol in the main chain or as side chains. The data related to the composition, molecular weight, and molecular weight distribution of polymers are presented. Moreover, other aspects, such as forms of carriers, types of encapsulated drugs, encapsulation efficiency and capacity, are also included.

## 1. Introduction

The inability to use the potential of available bioactive substances is an important issue of modern medicine. Many of the known drug molecules are successful when colliding with cells of bacteria, fungi, and tumors. However, their application in a conventional form is characterized by limited effectiveness due to low solubility, poor biodistribution, poor stability and rapid clearance from the body [1,2]. Therefore, smart drug delivery systems (DDS) are widely investigated and developed to improve the effectiveness of therapy by optimizing the dose and duration of drug action directly in the target site [1,3,4,5].

A variety of DDS, such as stimuli-responsive polymeric nanoparticles, liposomes, organic-inorganic hybrids, and exosomes, has been reported in scientific journals [6,7,8,9,10]. Among them, polymers are promising drug carriers because of the flexibility in the control of chemical compositions and functions of macromolecules (drug conjugation, stimulus sensitivity, stealth properties, specific targeting, etc.). A plethora of polymers has been used to obtain carriers with an innumerable variety of physicochemical and biological properties. The most frequently used are biocompatible and biodegradable polymers of natural or synthetic origin such as chitosan (CS) [11,12,13] hyaluronic acid (HA) [13,14,15,16,17], peptides [18], *N*-(2-hydroxypropyl)methacrylamide (HPMA) [19,20,21,22,23,24,25,26,27], poly(ethylene glycol) (PEG) [25,26,28,29,30,31,32,33,34,35,36,37,38,39,40,41,42,43,44,45,46,47,48,49,50,51,52,53,54,55,56,57,58,59,60,61,62,63,64,65,66,67,68,69,70,71,72,73,74,75], poly(glutamic acid) (PGA) [53,76,77], poly(lactic acid) (PLA) [28,78,79], and poly(d,l-lactide-co-glycolide) (PLGA) [29,53,80]. Their advantages are low toxicity, reduction of possible side effects, and ease of excretion [3,4,81].

**Figure 1:**
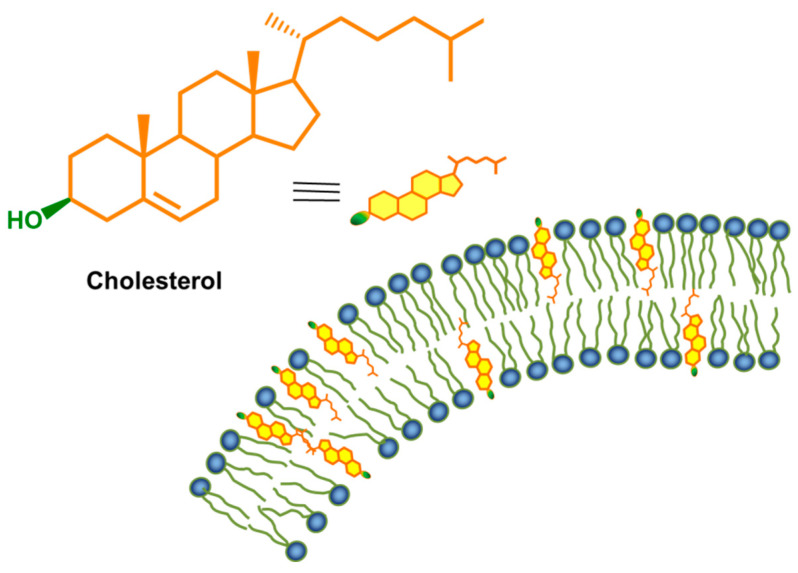
The structural formula of cholesterol and interaction in lipid bilayer.

One of the critical issues related to the efficiency of smart drug nanocarriers is their interaction with cell membranes. The modification of a carrier structure with a cell-penetrating ligand is a strategy to improve cellular uptake [82,83]. Cholesterol (Figure 1) is an organic compound, a steroid lipid, which is an essential structural component of animal cell membranes. It is responsible for the integration, fluidity, microdomain structure (so-called lipid rafts), and the permeability of the membrane. Cholesterol owes these properties to its structure—the hydroxyl group interacts with water molecules similar to the hydrophilic main groups of phospholipids, while the carbon skeleton shows a high affinity for the hydrophobic tails of phospholipids. The rigid and flat tetracyclic structure regulates the fluidity of the cell membrane [84,85]. Furthermore, cholesterol is a precursor in the biosynthesis of a wide range of biologically important substances, including bile acids, vitamin D, and sex hormones [86,87].

Due to its hydroxyl group which can easily be derivatized, large-scale availability, and relatively low cost, cholesterol has been used as a starting material for the synthesis of diverse steroid-based molecules [88,89]. High biocompatibility and the ability to be incorporated into cell membranes make cholesterol and its derivatives increasingly used in DDS.

In this review, we present an overview of numerous publications devoted to polymers comprising cholesterol designed for drug delivery. We describe the methods of incorporation of cholesterol moiety/ies into a polymer chain and the forms of drug carriers that have been obtained using these polymers. We present the types of drugs that have been encapsulated and the effectiveness (encapsulation efficiency and capacity) of their loading. Some examples of polymers bearing cholesterol moieties designed for drug delivery have been featured in more general review covering the topic of cholesterol chemistry and its applications in different research fields [88]. Moreover, there has been one earlier review concerning polymers comprising cholesterol, published in 2009 [90]. It was mainly focused on synthesis and strategies of direct ordering and packing of meso- and nanostructures of cholesterol polymers in the neat or melt state and in solution. It also dealt with their various applications, including drug delivery. However, the topic of drug delivery systems based on polymers comprising cholesterol has moved forward significantly since then.

## 2. Methods of Synthesis of Polymers Containing Cholesterol

**Figure 2:**
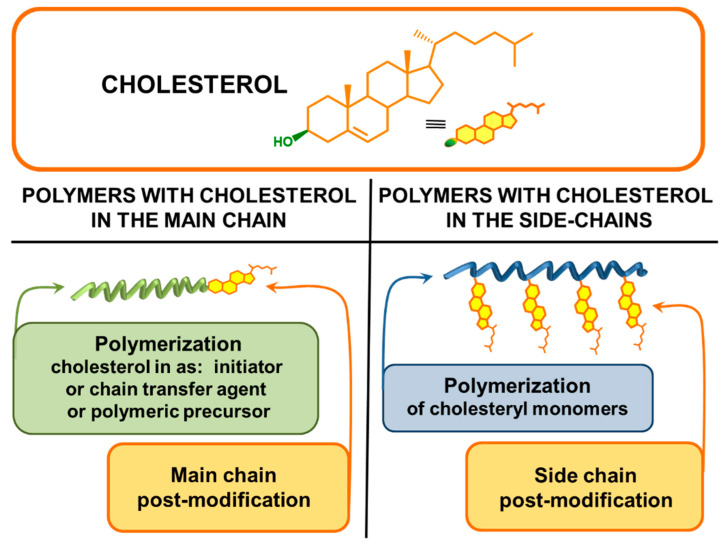
Methods of synthesis of polymers containing cholesteryl moieties.

A variety of polymerization methods and selective chemical reactions allow for the obtention of polymers with cholesterol incorporated both in the main chain and in side chains [88,90]. Due to the methodology of their preparation, polymers comprising cholesterol can be divided into polymers obtained (I) by polymerization of cholesterol-containing monomers, (II) by post-modification of side chains, (III) using a chain transfer agent or an initiator containing cholesterol, and (IV) as a result of chain end post-modification (Figure 2).

### 2.1. Polymers Containing Cholesterol in the Main Chain

**Figure 3:**
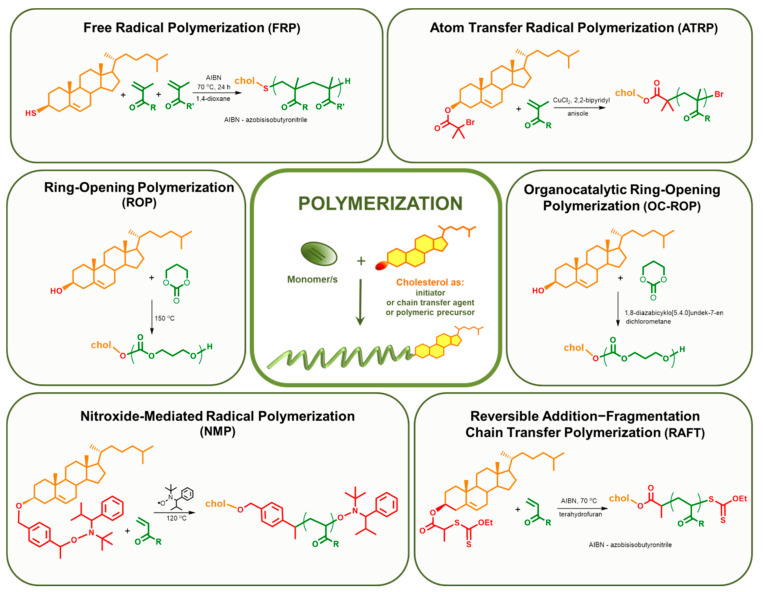
Polymerization methods used for the synthesis of cholesterol end-capped polymers and reaction examples [24,52,54,91,99,100,101].

**Figure 4:**
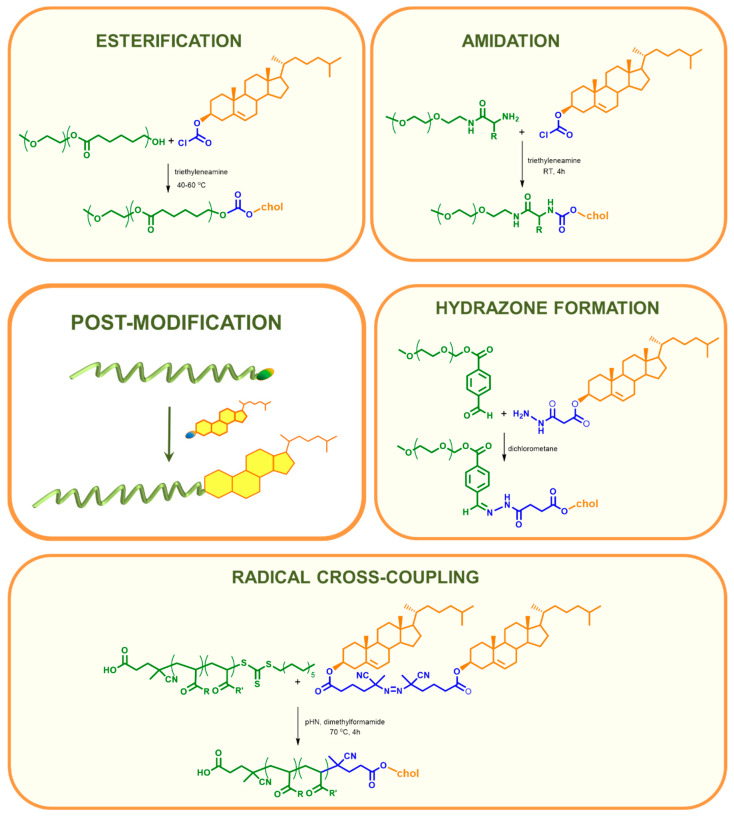
Post-modification reactions used to introduce cholesteryl moieties at the end of the polymeric chain [27,58,69,74].

There are two approaches that give the possibility to introduce cholesterol moiety at the end of the polymeric chain: the use of cholesterol-containing initiators or chain transfer agents (Figure 3, Table 1) and post-modification of the reactive chain end (Figure 4, Table 2). These approaches result in polymers with one cholesteryl moiety. In the context of drug delivery, recent studies [91] show that even with a relatively long polymer ballast, it is possible to take advantage of the properties of cholesterol present at the polymer chain end. In such systems, cholesterol plays primarily a guiding role, but it can also integrate into the biological membrane, which may allow releasing drug molecules in the immediate vicinity or even inside a pathological cell. Considering solubility in physiological fluids, one hydrophobic cholesterol moiety is an advantage, as such a system does not have to contain excessively large hydrophilic part to be soluble.

#### 2.1.1. Cholesterol Introduced to the Main Chain during Polymerization

Free radical polymerization (FRP) is the simplest method used in the synthesis of DDS. Due to the non-specific nature of free radicals, FRP is a versatile method that allows the polymerization of most vinyl monomers. The advantage of this type of polymerization is that it is not an expensive and fast method. Additionally, isolation and purification of the product are relatively easy. The significant disadvantages of FRP include high dispersity of the obtained systems and dead-end product, due to the termination processes, which preclude the copolymerization of subsequent blocks. The twentieth century was rich in the development of new polymerization methods, atom transfer radical polymerization (ATRP) in 1995 [92,93], and reversible addition–fragmentation chain transfer polymerization (RAFT) in 1998 [94]. With the growing interest in the field of drug delivery, polymeric carriers, star systems, dendrimers, and nanogels began to be used. Such structures are easier to obtain using CRP methods as compared to conventional FRP. Another advantage of controlled radical polymerization is the eventuality of obtaining a system with lower dispersity and the possibility of creating copolymer libraries that originate from one precursor polymer. For instance, in RAFT polymerization, there is a RAFT agent at the end of the chain, which can be re-initiated to propagate other blocks [95]. Additionally, functionalized monomers can be polymerized by CRP techniques. In the group of radical polymerizations with reversible deactivation (RDRP), the RAFT method has the greatest tolerance on reactive functional groups [95]. On the other hand, there is a multitude of types of ATRP, resulting in the high flexibility of this method [96]. CRP methods are not perfect and free from drawbacks, particularly when making use of the drug delivery systems thus obtained. Often, it is not possible to avoid the use of toxic initiators, which contain transition metals such as iron, copper, tin, or osmium (ATRP) [97]. In RAFT polymerization, it is necessary to use an additional factor which is the chain transfer agent (CTA), also called the RAFT agent. Dithiocarbonates and trithiocarbonates, which are used the most often, remain at the end of the polymer chain. This can also be a big disadvantage because these ends show toxic properties for the human body. However, at the same time, they allow for further copolymerization or appropriate modification, e.g., to the thiol group [98], which opens up a variety of possibilities from the Michael reaction to the formation of disulfides.

The majority of the methods used in the synthesis of polymeric drug delivery systems with an incorporated cholesterol molecule are controlled radical polymerizations such as ATRP [54,79,102,103,104,105,106,107,108], RAFT [91] or NMP [100] (Table 1). This is due to the possibility of controlling the dispersion of the system, which translates into stability in biological properties and accuracy in predicting the behavior of the carrier in the human body. Ring-opening polymerization techniques are also widely used, and most popular is the polymerization of cyclic monomers such as ε-caprolactone [109] and trimethylene carbonate [51,78,99].

#### 2.1.2. Cholesterol Introduced to the Main Chain by Post-Modification

The chemistry of polymers makes it possible to obtain functional macromolecules in a simple, fast, and relatively inexpensive way. However, in some cases, the presence of certain functional groups makes it impossible to perform polymerization. Post-polymerization modification, which is a combination of the achievements of polymer chemistry and organic synthesis, comes to the rescue. It consists of the preparation of a polymer that has modifiable, available groups, which are then subjected to various reactions from simple esterification (*O*-acylation) or amidation (*N*-acylation) through various coupling reactions to click reactions (Figure 4). The advantage of this methodology is the formation of functional products that are impossible to obtain by polymerization. The post-modification approach allows the creation of a library of functional polymers based on one reactive precursor, which ensures the maintenance of the same structural parameters such as tacticity, molecular weight distribution, or the degree of polymerization. In many cases, it turns out that the polymerization of a commercially available monomer and the subsequent functionalization of the polymer is a less time-consuming and cost-intensive method than the synthesis and polymerization of an original monomer. Additionally, the storage of the reactive monomer is at greater risk than the polymeric precursor [110,111]. On the other hand, it should be remembered that the post-polymerization approach has some important limitations. In the case of the functionalization of polymer precursors, it should be taken into account that organic reactions do not run with a 100% yield. This is caused by many factors, such as (I) availability of reactive groups; (II) possible steric hindrance in the polymer chain; (III) curling, twisting the polymer; or (IV) the need for additional purification, either from catalysts or other reactants used.

The introduction of cholesterol at the end of the polymer chain by post-modification occurs mainly through basic organic chemistry reactions such as esterification and amidation (Table 2). In this case, the simpler the better, thus reducing the time, cost, and risk of failure. The esterification of the OH-terminated polymer in dichloromethane in the presence of 4-dimethylaminopyridine (DMAP) with commercially available cholesteryl chloroformate and triethylamine is often used [58,112,114,116]. Reactions with succinyl cholesterol have been also reported, and, in these cases, dicyclohexylcarbodiimide (DCC) or 1-(3-dimethylaminopropyl)-3-ethylcarbodiimide (EDC) was used [56,57,59]. Amidation is mainly based on EDC coupling, where the NHS ester derivative of cholesterol or polymer is dissolved in an organic solvent (DCM, MeOH, or DMSO); then, in the presence of EDC, it reacts with a previously prepared amine-terminated derivative of a polymer or cholesterol, respectively [25,26,67,69,72,120]. There is also the possibility to introduce cholesterol in a radical cross-coupling [27] reaction or by hydrazone formation [73,74,75].

### 2.2. Polymers Containing Cholesterol as Side Chains

**Figure 5:**
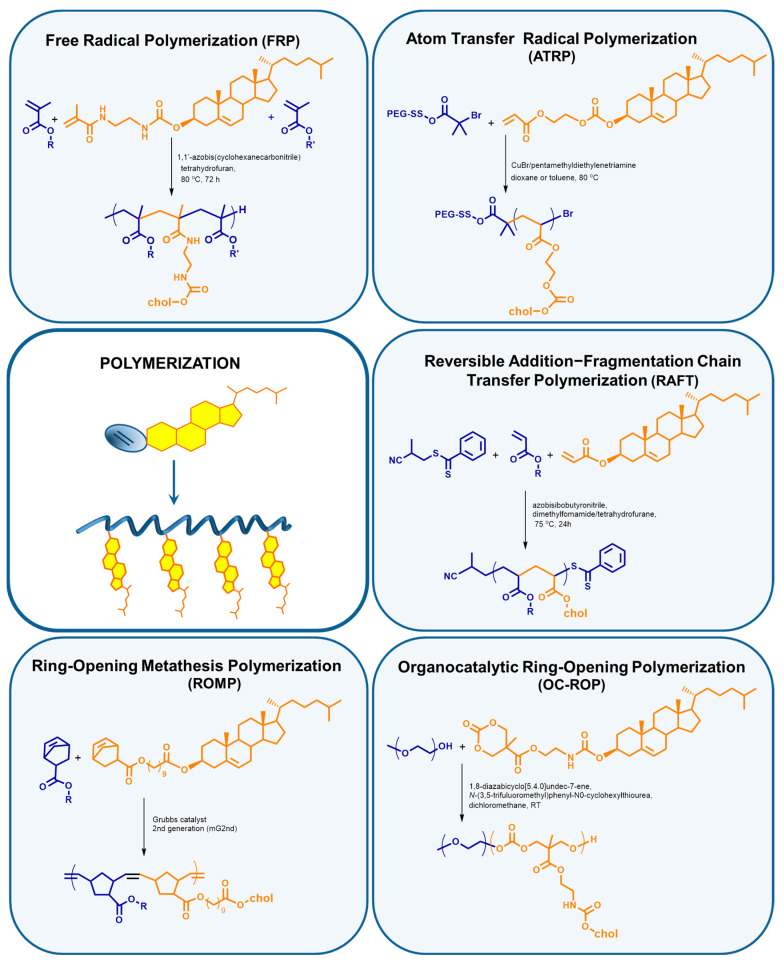
Types of polymerization used to obtain polymers with cholesterol moieties as side chains, and reaction examples [30,32,34,35,36].

**Figure 6:**
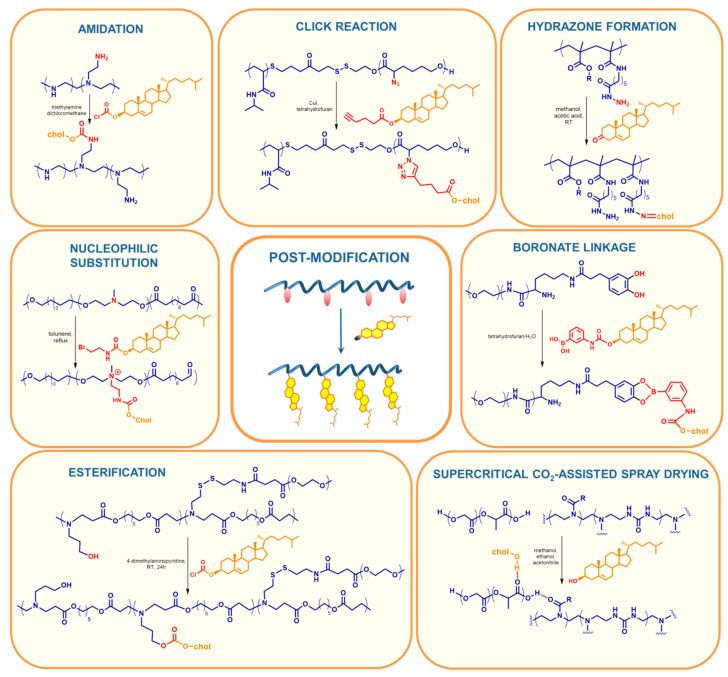
Methods of incorporation of cholesteryl moieties to side chains by post-polymerization reactions [20,38,39,80,130,132,133].

Similarly to the polymers containing cholesterol in the main chain, the incorporation of cholesterol to the side chains can occur by polymerization of a cholesterol-containing monomer (Figure 5, Table 3) or by post-polymerization modification (Figure 6, Table 4). Compared to cholesterol end-capped polymers, this approach allows the incorporation of multiple cholesterol molecules into a single polymeric chain. However, it carries a large ballast of the hydrophobic part, which has a negative effect on aqueous solubility. As a consequence, it creates the need to extend or add a new hydrophilic block (most often PEG), which in turn increases the weight of the carrier introduced into the body.

#### 2.2.1. Polymers Containing Cholesterol Moieties as Side Chains Obtained by Polymerization of Cholesterol-Based Monomers

Many different polymerization techniques are used to obtain polymeric drug carriers with cholesterol moieties as side chains, ranging from free radical polymerization to various types of controlled polymerization methods, such as RAFT, ATRP, and a variety of ring-opening polymerization methods such as ring-opening metathesis polymerization (ROMP) and organocatalytic ring-opening polymerization (OC-ROP) (Figure 5, Table 3). The variation of the methods used is due to many factors. One, as in all areas of life, is economics, i.e., the method should be non-expensive, technically simple, limit the use of toxic chemicals and give a clean product with high efficiency. However, in the case of DDS, it is not easy to achieve, because products that have a complex spatial structure and consist of many block-elements are considered. Drug delivery systems, due to their destination—the human body—should be characterized as accurately as possible. The more monodisperse sample, the more accurate its properties and expected behavior in the body. In the case of polymeric systems characterized by high dispersity, it is almost impossible to conclude the mechanism of action, metabolism, or removal. Therefore, the controlled polymerization techniques, which allow precise designing of polymers of desired molecular weight (number of repeating units), spatial structure, and low dispersion, are the methods of choice.

Similar copolymers of HPMA and various methacrylic cholesterol derivatives have been obtained by FRP or RAFT. The copolymer obtained by the controlled polymerization was characterized by significantly lower dispersion (1.39) [23] than the analogous copolymers produced by FRP (1.65–1.90) [19,20,21,22], despite the weight being approximately twice as high. The most important feature of ROPs is the ability to polymerize functionalized cyclic olefins [96]. However, it also carries a toxic ballast in the form of initiators or catalysts based on transition metals such as tin, ruthenium, or molybdenum in the ROMP [96].

#### 2.2.2. Polymers Bearing Cholesterol Moieties as Side Chains Obtained by Post-Modification

In addition to the classical amidation or esterification methods, which require the use of catalysts such as 1-(3-dimethylaminopropyl)-3-ethylcarbodiimide (EDC), dicyclohexylcarbodiimide (DCC), 4-dimethylaminopyridine (DMAP), or 1,8-diazabicyklo[5.4.0]undek-7-en (DBU), there are increasing possibilities of functionalization of polymers through post-modification, growing with the development of organic chemistry. The use of polymers in the drug delivery process requires the highest purity of polymer systems. Toxic catalysts and solvents, or complex and time- and cost-consuming purification processes force scientists to create new synthetic methods that take place under milder conditions. Alternative methods such as supercritical CO_2_-assisted spray drying (SASD) [80], nucleophilic substitution (Br to N) [129,130,131] or N-acylation [132] are gaining popularity due to the lack of catalysts and simple isolation and purification of reaction products (Figure 6, Table 4).

The major disadvantage of the post-modification approach is reaction efficiency (usually much below 1) and, as a consequence, a need to use an additional analytical method to determine the degree of post-modification, which increases costs and time of the process. The search for new organic reactions carried out under mild conditions (e.g., Michael addition [144] or orthogonal reactions [158]) and with high yield, the usage of magnetically separable catalysts [159] and the development of new and accurate methods of physicochemical analysis allow us to assume that the post-modification procedure will be further explored.

In the available scientific literature, there are many simplifications, which make it difficult to draw faultless conclusions. The complete physicochemical characterization of the final product is often missing. For instance, the molecular weight and/or dispersity index of the system after post-modification are not determined (Table 4).

## 3. Form of Carriers

**Figure 7:**
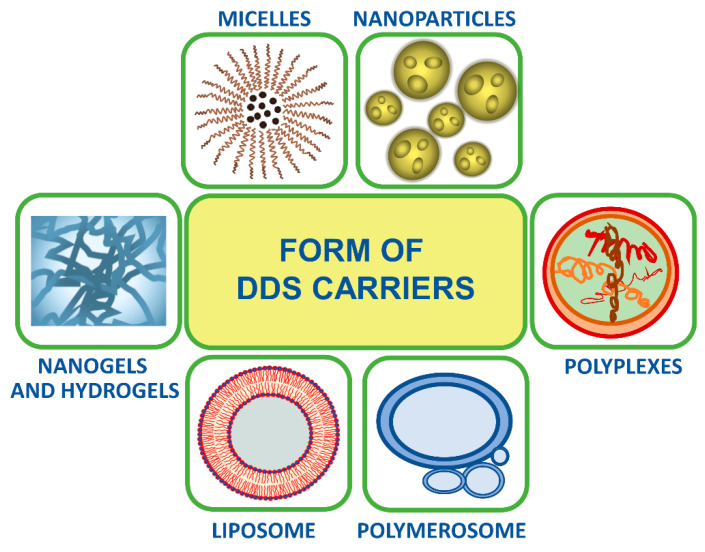
Forms of drug carriers obtained from polymers bearing cholesteryl moiety/ies.

There are various forms of drug carriers obtained from polymers bearing cholesteryl moiety/ies (Figure 7).

Micellar systems are the simplest constructs in the drug delivery area. They are made of amphiphilic copolymers, the major part of which is hydrophilic. The size of micelles ranges from 5 to 100 nm. These nanospheres are formed in thermodynamic conditions through self-assembly or with an additional factor. They are characterized by a critical micelle concentration (CMC), which is in the range of 10^−7^ to 10^−3^ M in water. At the appropriate concentration, the micelles may disintegrate and return to be unimers [160], which may be both the advantage or disadvantage depending on their application. The most common method to obtain micelles used in drug delivery is the precipitation method, where the appropriate selection of the conditions (temperature, concentration, solvent, or their mixture) plays a key role in loading efficiency [161]. During the formation of micelles, it is also possible to encapsulate active substances, which are mostly hydrophobic compounds [162].

Nanoparticles (NPs) are nanostructures made of amphiphilic copolymers with predominated lipophobic part, which are prepared under kinetic conditions. Their sizes are in the range of 50–200 nm. NPs are characterized by higher colloidal stability than micelles, and they do not decay into unimers. In drug delivery, they protect against coagulation, aggregation, or phagocytosis, and especially in the case of core/shell type nanoparticles, the core is responsible for the transport of the hydrophobic drug, while the shell acts as a shield and may have a guiding function [160].

Liposomes, i.e., phospholipid vesicles, are spherical structures made of a lipid bilayer. Due to the trapping of water inside the structure, they play the role of transporters of hydrophilic substances in living organisms. Similar structures—polymersomes—can be formed by polymeric amphiphilic compounds, in which the hydrophilic part is in the range of 20–40 wt. %. As liposomes, they are dedicated to transporting hydrophilic substances. Their sizes vary from 100 to 1000 nm [163]. One of the methods of producing polymersomes is the film rehydration method [164]. In the literature, there are examples of liposomes composed, inter alia, of cholesterol and other lipids, which are the building block of the double membrane and are not covalently bonded to the polymeric drug carrier. In such a case, the liposome is just a frame or a transporter of the proper working system. Constructing such systems is widespread due to high durability, simplicity of preparation and easy-to-predict behavior [165,166,167,168,169].

Polymer gels are a three-dimensional network of polymer chains, which is formed by chemical or physical cross-linking. A specific group is hydrogels, which are insoluble in water and do not lose their structural integrity, even in the case of high water concentration. Due to their high water absorption, even over 90 wt. %, they are sorbents with great use, for example, in diapers. Hydrogels that occur in the form of nanoparticles are called nanogels. They have diameters of tens to hundreds of nanometers. These are porous materials that can be filled with, for example, drug molecules. It is possible to design properties of hydro and nanogels, such as swelling, degradation, and chemical functionality by the use of various biopolymers or synthetic polymers as well as various cross-linking methods [170,171,172,173,174].

Nanoparticles that are formed by self-assembly of cationic polymers and DNA or RNA are called polyplexes. Such materials are capable of transporting exogenous genetic material into cells in a process called transfection [175].

Cholesterol has many functions in polymeric drug carriers. Its role in cellular uptake is crucial, and cholesterol-containing polymers are characterized by increased cellular uptake in the endocytotic pathway. The mode of action is not clearly described due to the differences in the spatial structure of carriers, encapsulated drugs and pathological targets. Cholesterol stimulates cellular uptake in a lipid rafter-dependent manner [67], by activating the low-density lipoprotein (LDL) receptor [38] and by interacting with glycosphingolipid-rich microdomains in the plasma membrane [127]. Cholesteryl moiety acts as a cell-penetrating agent that stiffens the membrane by embedding into it, which leads to membrane disintegration and tumor growth inhibition [91]. Additionally, the use of cholesterol drug carriers results in higher cellular uptake of the drug. The use of the same dose of loaded drug as the free drug leads to increased apoptosis of neoplastic cells [38,136]. Cholesterol may also function as complexing agent of hydrophobic drugs [76]. Still, there is a gap in the literature on the effect of the number of cholesterol groups in the polymer chain on the stability of plasma membranes and drug delivery.

## 4. Drug Encapsulation and Release

Drug loading capacity (DLC or LC) and drug encapsulation efficiency (DEE or EE) are the basic and most frequently determined parameters in drug delivery and applications. They are expressed as a percentage of the amount of drug-loaded per carrier weight or the amount of drug effectively entrapped in the carrier, respectively. The EE can be calculated as the total weight of the entrapped drug divided by the total weight of the drug added, while the LC is the quotient of the total weight of the entrapped drug and the total weight of the drug-loaded carrier.

$$\mathrm{EE}\;(\%)=\frac{\mathrm{total}\;\mathrm{weight}\;\mathrm{of}\;\mathrm{the}\;\mathrm{entrapped}\;\mathrm{drug}}{\mathrm{total}\;\mathrm{weight}\;\mathrm{of}\;\mathrm{drug}\;\mathrm{added}}\times 100$$

$$\mathrm{LC}\;(\%)=\frac{\mathrm{total}\;\mathrm{weight}\;\mathrm{of}\;\mathrm{the}\;\mathrm{entrapped}\;\mathrm{drug}}{\mathrm{total}\;\mathrm{weight}\;\mathrm{of}\;\mathrm{the}\;\mathrm{drug}-\mathrm{loaded}\;\mathrm{carrier}}\times 100$$

These parameters depend on many factors, including the mass ratio of drug to a vehicle; the method of preparing micelles, nanoparticles, liposomes, and other forms; composition, architecture and arrangement of the polymeric carrier; size and functional groups of the drug molecule; the number of functional groups in the carrier that can complex the drug; the type of drug-carrier interactions; the tendency of polymer chains to twist and self-organize; and the time of dialysis and frequency of water changes.

The variable mass ratio of drug to vehicle is the most frequently studied and easiest to perform the comparison. The same procedure is carried out only by changing the amount of the drug, and the results of such studies show that the research problem is complex. In most cases, as the number of drug increases, EE decreases with increasing LC values [12,29,57,72,136], and, in some cases, both LC and EE values increase [41,152]. It seems logical that by increasing the mass of the added drug, we increase the final loaded mass of the drug in the carrier, but one should consider whether the limit is. The results of the research [29,69] show that there is a critical point at which the maximum value of loading capacity of the DDS is reached, and at some point, there is a drastic decrease in EE from>95% to about 70%, while the LC increase is around 1%.

The preparation of nanoparticles has a huge impact on loading capacity and encapsulation efficiency, with solvent selection, temperature, and dialysis time, playing an important role. A solvent, in which both the drug and polymer have the best solubility and can be easily removed without destroying the structures formed, should be selected. A common choice is DMSO or DMF as well as other volatile organic solvents [113]. Dialysis at elevated temperature often leads to an increase in EE [113], while prolonged time reduces both EE and LC values [11].

An effort is being made to determine the relationship between the structure of a carrier and a drug molecule by studies on loading different molecules into the same carrier [15,19,53], and there are also reports on co-loading. It is a very individual matter, and, to date, due to the complexity of the problem, it has not been possible to find a clear answer as to which factors determine the effectiveness of loading. Co loading reduces the LC value [17,55,71], however, it may positively influence EE [17].

Considering the composition of polymeric drug carriers containing a cholesteryl (Chol) moiety, a relevant parameter is the ratio of hydrophilic to hydrophobic parts. The addition of hydrophobic block lowers EE and LC values, whereas hydrophilic units such as PEG [44], folic acid (FA) [136], poly(ε-caprolactone) (PCL) [58], or histidine (HIS) [153], raise these parameters significantly.

The influence of the cholesterol content in the carrier does not translate unequivocally into the encapsulation efficiency or loading capacity [48,128,149,154]. On the other hand, a positive effect is exerted by the change of the carrier’s architecture from linear to dendrimer, which raises both the EE and LC values [70].

In vivo release kinetics studies have been described in many articles. Due to the different local environment of neoplastic cells and normal cells, in particular, the slightly acidic environment within neoplastic cells, research is conducted in order to obtain a pH-sensitive carrier that will release drug molecules at a pH below 6. The conducted research indicates that drug delivery systems not only enable the control of the release depending on pH, but also slow down the release of the active substance over time, which reduces toxicity and reduces the side effects of the used therapies [11,19,20,112,146]. Most of the systems in which cholesterol is covalently bound to the polymer chain by groups are easily hydrolyzed under physiological conditions, and the products of metabolism are an important aspect of the research. Steroid groups ensure the biocompatibility of polymeric carriers and reduce the toxicity of such systems on normal cells [91]. Chytil et al. investigated the amount of cholesterol derivatives released from hydrazone bonding systems. Despite the hydrolysis, the systems were characterized by low toxicity and, at the appropriate pH, a high percentage of drug molecules released [20].

## 5. Conclusions

The number of publications that have emerged in recent years shows huge potential hidden in the use of natural products in medical applications. The challenges in the field of drug delivery (specific targeting, intracellular delivery, stimuli-controlled release, etc.) may be met by the application of systems based on polymers containing steroids or their derivatives. Due to the high availability, relatively low price, and hydroxyl group that can easily be derivatized, cholesterol is mainly used for this purpose. The introduction of cholesteryl groups into the structure of the carrier improves its biological properties, biocompatibility, and biodistribution. Polymer chemistry and organic chemistry are developing rapidly, which increases the synthetic possibilities and enables the formation of more complex and more effective systems. Both approaches, polymerization of functional monomers and post-modification, have been successful in the synthesis of polymeric drug carriers containing cholesterol moiety/ies in the main chain or as side chains. Both have advantages and disadvantages that should be taken into account in the course of designing and preparing DDS. Certainly, the molecular weight and molecular weight distribution of the system are factors that have a significant impact on its behavior in the biological environment, and, hence, they should be accurately defined. In this regard, various controlled radical polymerization techniques are the methods of choice as they provide polymers with well-defined properties. In the case of post-modification, the efficiency of the reaction should be taken into account, as it has a huge impact on the properties of the system. The use of diverse polymers and possibility of their modification allows the encapsulation of almost any substance. Polymeric drug carriers containing cholesterol in their structure are mainly tested for the transport of anti-cancer [16,17,48,70,71,72], anti-fungal [58,152], antibacterial [117], and anti-inflammatory drugs [44,99,117] as well as antioxidants [15,54,117]. By using cholesterol-containing systems, endocytosis or fusion of siRNA [100] or pDNA [134] is possible. It is worth noting that there are promising studies on the transmembrane transport of cholesterol-modified siRNA [176].

## Figures and Tables

**Table 1:** Cholesterol end-capped polymers reported as drug delivery systems.

Polymer	Form of Carrier	Drug or Dye	M_n_ or M_w_ (kDa) (Ð)	LE (%)	LC (%)	Lit.
**Free Radical Polymerization (FRP)**						
Chol-pHPMAlac (mono:di = 30:70)	liposome	DOX	10.5 (1.60–1.70)	93.0	N/A	[24]
Chol-pHPMAlac (mono:di = 44:56)			10.0 (1.60–1.70)	99.0		
Chol-pHPMAlac (mono:di = 54:46)			11.0 (1.60–1.70)	100		
Chol-pHPMAlac (mono:di = 67:33)			11.0 (1.60–1.70)	100		
**Nitroxide-Mediated Controlled Radical Polymerization (NMP)**						
Chol-PAA	PCLp	siRNA	N/A	46.0	0.8	[100,101]
**Atom Transfer Radical Polymerization (ATRP)**						
Chol-PDMAEMA	liposome	CF	5.4 (1.17)	N/A	N/A	[102]
		calcein				
Chol-PAA 5% in lip	liposome	calcein	7.2 (N/A)	29.6	N/A	[103,104]
Chol-PAA 10% in lip				46.1		
Chol-PAA 20% in lip				28.8		
Chol-PAA 10% in lip crosslinked				24.7		
Chol-LC-PDMAEMA	liposome	calcein	N/A	N/A	N/A	[105]
Chol-PLA-SS-PMPC	micelle	Nile red	N/A	N/A	N/A	[79]
Chol*-b-*pMPC	polymersome	ADR	6.4 (N/A)	3.6	N/A	[106]
			9.5 (N/A)	4.2		
			15.4 (N/A)	4.0		
	micelle	ADR	3.0 (N/A)	N/A	N/A	[107]
			6.4 (N/A)			
			N/A			
Chol-PEO	micelle	ADR	1.7 (1.13)	N/A	10.1	[108]
			2.3 (1.10)		16.2	
			2.8 (1.10)		16.9	
Chol-PEGMA_50_	micelle	QC	33.2 (1.25)	N/A	15.6	[54]
Chol-PEGMA_100_			52.2 (1.32)		14.1	
Chol-PEGMA_200_			89.1 (1.55)		14.1	
**Reversible Addition–Fragmentation Chain Transfer Polymerization (RAFT)**						
Chol-PNIPAAm	micelle	N/A	3.2 (1.27)	N/A	N/A	[91]
			5.7 (1.35)			
			6.1 (1.51)			
			8.4 (1.64)			
			10.9 (1.90)			
**Ring-Opening Polymerization (ROP)**						
Chol-PCL (nChol:nPCL = 1:4)	nanoparticle	prednisone acetate	2.0 (1.49)	N/A	N/A	[109]
Chol-PCL (nChol:nPCL = 1:10)			5.5 (1.34)			
Chol-PCL (nChol:nPCL = 1:20)			7.2 (1.55)			
Chol-PCL (nChol:nPCL = 1:40)			11.4 (1.69)			
Chol-PCL (nChol:nPCL = 1:80)			16.2 (1.79)			
Chol-pTMC (nChol:nTMC = 1:4)	nanoparticle	prednisone acetate	1.8 (1.26)	N/A	N/A	[99]
Chol-pTMC (nChol:nTMC = 1:10)			2.7 (1.75)			
Chol-pTMC (nChol:nTMC = 1:20)			5.2 (1.78)			
Chol-pTMC (nChol:nTMC = 1:40)			9.7 (1.65)	61.7	9.1	
Chol-pTMC (nChol:nTMC = 1:80)			13.9 (1.80)	N/A	N/A	
**Organocatalytic Ring-Opening Polymerization (OC-ROP)**						
Chol-PTMC-PEG	nanoparticle	DOX	6.6 (N/A)	N/A	7.3	[52]
Chol-PTMC	surface	FITC-BSA	11.3 (1.20)	N/A	N/A	[78]
Chol-PTMC-PLA			10.1 (1.40)			
Chol-PTMC-PMBC			2.5 (1.20)			
Chol-PMBC			3.3 (1.50)			

Abbreviations: ADR, Adriamycin; ATRP, atom transfer radical polymerization; Chol, cholesterol; CF, 5,6-carboxyfluorescein; DMAEMA, 2-(dimethylamino)ethyl methacrylate; DOX, doxorubicin; FITC-BSA, fluorescein isothiocyanate-labeled bovine serum albumin; LC, lecithin; MBC, 5-methyl-5-benzylcarboxyl-1,3-dioxan-2-one; MPC, 2-methacryloyloxyethyl phosphorylcholine; NIPAAm, *N*-isopropylacrylamide; OC-ROP, organocatalytic ring-opening polymerization; PAA, poly(acrylic acid); PCL, poly(ε-caprolactone); PCLp, polymer-caged lipoplex; PEG, poly(ethylene glycol); PEGMA, poly(ethylene glycol) methyl ether methacrylate; PEO, poly(ethylene oxide); PHPMAlac, poly(*N*-(2-hydroxypropyl)methacrylamide mono/dilactate); PLA, poly(lactic acid); RAFT, reversible addition−fragmentation chain transfer polymerization; ROP, ring-opening polymerization; TMC, trimethylene carbonate; QC, quercetin.

**Table 2:** Cholesterol end-capped polymers obtained by post-modification reported as DDS.

Polymer	Form of Carrier	Drug or Dye	M_n_ or M_w_ (kDa) (Ð)	LE (%)	LC (%)	Lit.
**Esterification**						
Acetylene-PEG_10K_-G_4_-Chol_16_	micelle	DOX	14.2 (1.11)	N/A	18.8	[55]
		TPL			8.4	
		DOX + TPL			N/A/6.5	
Rhodamine-PEG_10K_-G_4_-Chol_16_		DOX	17.6 (1.14)	N/A	10.0	
mPEG-Chol	micelle	DTXL	N/A	97.6	4.8	[56]
mPEG-Chol/RGD-mPEG-Chol (10% mPEG-Chol *w*/*w*)	liposome	PTX (2.5% *w*/*w*)	N/A	99.8	0.05	[57]
		PTX (5% *w*/*w*)		99.6	0.08	
		PTX (7.5% *w*/*w*)		99.1	1.15	
		PTX (10% *w*/*w*)		97.3	1.62	
mPEG-Chol/RGD-mPEG-Chol (20% mPEG-Chol *w*/*w*)		PTX (2.5% *w*/*w*)		99.7	0.53	
		PTX (5% *w*/*w*)		99.1	0.81	
		PTX (7.5% *w*/*w*)		98.9	1.06	
		PTX (10% *w*/*w*)		95.1	1.48	
mPEG-Chol	micelle	AmB	5.9 (1.04)	42.0	8.8	[58]
mPEG*-b-*PCL-Chol			10.1 (1.20)	60.0	12.5	
TPGS-Chol	micelle	DTXL	N/A	99.2	3.2	[112]
Chol-PEG-GA	liposome	brucine	N/A	82.5	N/A	[59]
Chol-PEG_2K_/(γ-PGA*-g-*PLGA)	nanoparticle	DOX	N/A	63.8	4.6	[53]
Chol-PEG_5K_/(γ-PGA*-g-*PLGA)				66.8	4.8	
Chol-PEG_10K_/(γ-PGA*-g-*PLGA)				66.6	4.7	
Chol-PEG_2K_/(γ-PGA*-g-*PLGA)		ICG		86.8	6.2	
Chol-PEG_5K_/(γ-PGA*-g-*PLGA)				86.8	6.2	
Chol-PEG_10K_/(γ-PGA*-g-*PLGA)				84.9	6.1	
PF127-Chol	micelle	DTXL (temp., ratio, solvent)	N/A	81.0	N/A	[113]
FA-PF127-Chol				65.4–103.2		
Chol-PSO	micelle	PTX	2.4	80.1	18.6	[114]
Chol-PSO-(HE)_5_-Fmoc/Chol-PSO-(RG)_5_-Pbf			N/A	78.5	17.1	
F68-Chol	micelle	CABA	N/A	98.1	3.2	[115]
mPEG-Chol	micelle	QC	N/A	93.5	3.7	[60]
Biotin-PAE*-g-*mPEG-Chol	micelle	DOX	11.8 (1.60)	61.0	5.5	[61]
PAE*-g-*mPEG-Chol			N/A	47.0	4.2	
mPEG–PLA-Chol	micelle	CUR	N/A	93.7	11.9	[28]
PEG-PLLA-Chol	micelle	DOX	N/A	45.3	8.3	[62,63]
PEG-PDLA-Chol				48.2	8.8	
Chol-PEG	micelle	PTX	N/A	>90	N/A	[64]
Chol–PEG–DUP1	micelle	PTX	N/A	96.4	24.9	[65]
Chol-mPEG-RGD/mPEG-PLGA	nanoparticle	CUR (2% *w*/*w*)	N/A	100	2.00	[29]
		CUR (3% *w*/*w*)		98.7	2.96	
		CUR (4% *w*/*w*)		97.8	3.91	
		CUR (5% *w*/*w*)		96.0	4.80	
		CUR (7% *w*/*w*)		70.7	4.95	
P(NIPAAm-*co-*DMAAm)*-g-*Chol	micelle	Py	2.9 (1.20)	N/A	0.8 mg/g	[116]
P(NIPAAm-*co-*DMAAm)*-g-*Chol			6.4 (1.30)		1 mg/g	
Chol-PEG-TPP	liposome	CF	N/A	1.8	N/A	[66]
mPEG-*b*-PCL-Chol	micelle	CUR	6.6 (1.17)	32.0	8.8	[117]
**Amidation**						
Chol−PEG−PpIX	micelle anchored to liposome	itself	N/A	N/A	N/A	[67]
HA–SA–CYS–Chol	micelle	DTXL	30.1 (1.70)	89.7	4.8	[16]
HA-Chol	nanoparticle	DTXL	N/A	66.9	1.9	[17]
		TMX		76.5	4.1	
		DTXL/TMX		83.1/92.5	1.4/3.4	
PEG-PAsp(DET)-Chol	micelle	pDNA	N/A	N/A	N/A	[68]
DMEDA-HPbCD-Chol:Pluronic F127	polyplex	siRNA	N/A	N/A	N/A	[118]
DMEDA-HPbCD-Chol:Pluronic L81						
DMEDA-HPbCD-Chol:Pluronic L35						
PAMAM-Chol	micelle	RES	N/A	N/A	46.5	[119]
PEG-Chol-α-TOC	micelle	CUR (5% *w*/*w*)	N/A	97.2	4.6	[69]
		CUR (10% *w*/*w*)		98.4	8.4	
		CUR (15% *w*/*w*)		98.6	14.2	
		CUR (20% *w*/*w*)		74.3	15.2	
mPEG*-b-*PEP*-g-*Chol) linear	micelle	DOX	5.8 (1.45)	42.1	15.7	[70]
mPEG*-b-*PEP*-g-*Chol) Y-shape			6.0 (1.33)	50.4	20.2	
mPEG*-b-*PEP*-g-*Chol) Fork-shape			6.5 (1.48)	58.5	23.1	
mPEG*-b-*PAMAM-G_1_-Chol_1_	micelle	DOX	5.3 (N/A)	40.0	4.7	[71]
		PTX		5.4	0.7	
		DOX/PTX		38.1/6.5	4.3/0.7	
mPEG*-b-*PAMAM-G_2_-Chol_2_		DOX	5.9 (N/A)	40.7	4.8	
		PTX		7.2	0.8	
		DOX/PTX		38.4/6.9	4.2/0.8	
mPEG*-b-*PAMAM-G_4_-Chol_4_		DOX	7.2 (N/A)	40.2	4.8	
		PTX		8.3	1.0	
		DOX/PTX		34.5/8.3	3.9/1.0	
mPEG*-b-*PAMAM-G_8_-Chol_8_		DOX	N/A	40.1	4.7	
		PTX		18.2	2.2	
		DOX/PTX		36.8/19.4	4.2/2.2	
Chol-P(HEMA-Lys)	liposome	siRNA	N/A (1.20)	N/A	N/A	[120]
mPEG-P(HPMA*-g-*His)-Chol	liposome	DOX	12.3 (1.06)	81.3	18.2	[25,26]
(Chol-PLGVRK-PEG):(DUPA-PEG-Chol) = 1:9	micelle	CABA (25% *w*/*w*)	N/A	79.7	12.0	[72]
		CABA (200% *w*/*w*)		38.9	43.8	
Chol*-g-*uPA-PAA	liposome	CF	N/A	N/A	N/A	[121]
CS*-g-*Chol*-g-*FA	micelle	PTX (4h dialysis)	N/A	75.6	12.9	[11]
		PTX (8h dialysis)		63.1	10.5	
		PTX (12h dialysis)		56.5	7.4	
		PTX (24h dialysis)		32.7	5.5	
Chol-DP7	micelle	itself	N/A	N/A	N/A	[122]
**Radical Cross-Coupling**						
p(HPMA-*r*-NAS)-Chol	polyplex	siRNA	17.7 (1.40)	N/A	N/A	[27]
p(HPMA-*r*-AEDA)-Chol			24.7 (1.20)			
p(HPMA-DMAE-*r*-AEDA)-Chol			34.1 (1.30)			
**Hydrazone Formation**						
mPEG-Hz-Chol	liposome	Arctigenin	N/A	93.8	N/A	[73]
		GEM	2.6 (N/A)	37.0	4.0	[74,75]

Abbreviations: AEDA, 2-((2-azidoethyl) disulfanyl) ethan-1-amine hydrochloride; AmB, Amphotericin B; CABA, cabazitaxel; Chol, cholesterol; CF, 5,6-carboxyfluorescein; CS, chitosan; CUR, curcumin; CYS, cystamine; DMAE, 2-(dimethylamino)ethyl 1H-imidazole-1-carboxylate; DMAAm, *N*,*N*-dimethylacrylamide; DMEDA, *N*,*N*-dimethylaminoethylamine; pDNA, plasmid DNA; DOX, doxorubicin; DP7, antimicrobial peptide (VQWRIRVAVIRK); DTXL, docetaxel; DUP1, peptide (CFRPNRAQDYNTN); DUPA, 2-[3-(1,3-dicarboxypropyl) ureido]pentanedioic acid; F68, Pluronic F68; FA, folic acid; Fmoc, 9-fluorenylmethoxycarbonyl; GA, glutamic acid; GEM, gemcitabine; HA, hyaluronic acid; (HE)_5_, histidine-glutamic acid decapeptide; HEMA, hydroxyethyl methylacrylate; HIS, histidine; HPbCD, modified 2-hydroxypropyl-b-cyclodextrin macrocycles; HPMA, *N*-(2-hydroxypropyl) methacrylamide; Hz, hydrazone; ICG, indocyanine green; Lys, lysine; mPEG, (poly(ethylene glycol) methylether methacrylate; NAS, *N*-acryloxysuccinimide; NIPAAm, *N*-isopropylacrylamide; PAA, poly(acrylic acid); PAE, poly(β-amino ester); PAMAM, polyamidoamine; PAsp(DET), poly{*N*-[*N*-(2-aminoethyl)-2-aminoethyl]aspartamide}; Pbf, 2,2,4,6,7-pentamethyldihydrobenzofuran-5-sulfonyl; PCL, poly(ε-caprolactone); PDLA, poly(d-lactide acid); PEG, poly(ethylene glycol); PEP, peptide; PF127, Synperonic PE/F 127; PGA, poly(glutamic acid); PLA, poly(lactic acid); PLGA, poly(d,l-lactide-co-glycolide); PLGVRK, matrix metalloproteinase-2 responsive peptide; PLLA, poly(l-lactide acid); PplX, protoporphyrin IX; PSO, polyoxyethylene sorbitol oleate; PTX, paclitaxel; Py, pyrene; QC, quercetin; RES, resveratrol; (RG)_5_, arginine-glycine decapeptide; RGD, arginylglycylaspartic acid; SA, succinic anhydride; TMX, tamoxifen; TPGS, tocopheryl poly(ethylene glycol) succinate; TPL, triptolide; TPP, triphenylphosphine; α-TOC, α-tocopherol; uPA, short peptide sequence for urokinase plasminogen activator.

**Table 3:** Polymers bearing cholesterol in side chains reported as drug delivery systems.

Polymer	Form of Carrier	Drug or Dye	Mn or Mw (kDa) (Ð)	LE (%)	LC (%)	Lit.
**Free Radical Polymerization (FRP)**						
mPEG-Chol-DMA (nChol:nDMA = 1:7)	polymersome	FITC-CM-Dex	N/A	60.0	N/A	[30]
mPEG-Chol-DMA (nChol:nDMA = 1:3)				59.0		
mPEG-Chol-DMA (nChol:nDMA = 1:1)				N/A		
mPEG-Chol-DMA (nChol:nDMA = 3:1)						
mPEG-Chol						
**Atom Transfer Radical Polymerization (ATRP)**						
PEG-SS-PAECChol	polymersome	Calcein	6.7 (1.14)	68.0	5.5	[35]
PEG*-b-*PAECChol			6.0 (1.13)	74.0	6.0	
**Reversible Addition–Fragmentation Chain Transfer Polymerization (RAFT)**						
P(AChol_15_-*co*-mPEG_5,110_)	micelle	CPT	39.0 (1.44)	N/A	5.5	[36]
P(AChol_3_-*co*-mPEG_23,22_)			25.0 (1.26)	N/A	3.5	
P(CholDEGA*-b-*(AAA-*r*-BnAAA)) (52% hydrogenated)	micelle	Nile red	N/A	25.0	N/A	[123]
P(CholDEGA*-b-*(AAA-*r*-BnAAA)) (70% hydrogenated)				25.0	N/A	
P(CholDEGA*-b-*(AAA-*r*-BnAAA)) (85% hydrogenated)				5.0	N/A	
P(CholDEGA*-b-*(AAA-*r*-BnAAA)) (52% hydrogenated)		IBU		>40	>25	
P(CholDEGA*-b-*(AAA-*r*-BnAAA)) (70% hydrogenated)				>30	>25	
P(CholDEGA*-b-*(AAA-*r*-BnAAA)) (85% hydrogenated)				>15	>10	
PLL(PMA-co-MAChol)	liposome	PTX	33.0 (1.05)	N/A	N/A	[124,125]
P(MAA-*co*-MAChol) (2 mol% chol)	nanocomplex	DOX	16.5 (1.19)	N/A	N/A	[126,127]
P(MAA-*co*-MAChol) (4 mol% chol)			15.8 (1.10)			
P(MAA-*co*-MAChol) (8 mol% chol)			18.0 (1.11)			
P(MAgala_18_*-b-*MAChol_14_)	micelle	DOX	12.8 (1.26)	47.1	10.5	[128]
P(MAgala_18_*-b-*(MAA_5_-*co*-MAChol_14_))			N/A	61.5	13.3	
P(MAgala_18_*-b-*(MAA_16_-*co*-MAChol_12_))				81.9	17.0	
P(MAgala_18_*-b-*(MAA_26_-*co*-MAChol_9_))				91.2	18.6	
P(HPMA-*co*-MA-εAhx-NHNH_2_-*co*-MA-εAhx-Chol)	nanoparticle	DOX	50 (1.39)	N/A	6.0	[23]
**Organocatalytic Ring-Opening Polymerization (OC-ROP)**						
mPEG_113_*-b-*P(MTC-Chol)_4_	micelle	N/A	7.5 (1.12)	N/A	N/A	[31]
mPEG_113_*-b-*P(MTC-Chol)_11_			11.8 (1.21)			
mPEG_113_*-b-*P(MTC-Chol_11_)	nanoparticle	PTX	11.8 (1.21)	N/A	3.8	[32]
mPEG_113_*-b-*P(MTC-Chol_8_-*co*-TMC_8_)			10.7 (1.18)		9.2	
mPEG_113_*-b-*P(MTC-Chol_11_-*co*-TMC_30_)			14.8 (1.20)		15.0	
mPEG_113_*-b-*P(MTC-Chol_18_-*co*-TMC_55_)			21.7 (1.17)		8.4	
**Ring-Opening Metathesis Polymerization (ROMP)**						
P(NBChol*-b-*NBmPEG)	nanoparticle	DOX	162 (1.30)	58.0	14.5	[33]
P(NBChol)_50_*-b-*(NBmPEG)_170_	nanoparticle	DOX	126 (1.24)	88.4	22.1	[34]
P(NBChol)_75_*-b-*(NBmPEG)_255_			216 (1.16)	68.8	17.2	
P(NBChol)_180_*-b-*(NBmPEG)_222_			118 (1.16)	79.2	19.8	

Abbreviations: AAA, ascorbyl acrylate; AChol, cholesteryl acrylate; AECChol, cholesteryl acryloyoxy ethyl carbonate; ATRP, atom transfer radical polymerization; BnAAA, benzyl protected ascorbylacrylate; Chol, cholesterol; CholDEGA, cholesteryl diethyleneglycol acrylate; CPT, S-(+)-camptothecin; DMA, 1-decyl methacrylate; DOX, doxorubicin; DTXL, docetaxel; FITC-CM-Dex, fluorescein isothiocyanate carboxymethyl dextran; HIS, histidine; HPMA, *N*-(2-hydroxypropyl)methacrylamide; IBU, ibuprofen; MAA, methacrylic acid; MA-ɛAhx-Chol, cholest-5-en-3β-yl 6-methacrylamido hexanohydrazide; MA-ɛAhx-Chol_43_, cholest-4-en-3β-yl 6-methacrylamido hexanohydrazide; MA-ɛAhx-Chol_5α_, 5α-cholestan-3β-yl 6-methacrylamido hexanohydrazide; MA-ɛAhx-NHNH2, 6-methacrylamido hexanohydrazide; MA-ɛAhx-LevChol, cholest-5-en-3β-yl 4-oxopentano 6-methacrylamido hexanohydrazide; MA-ɛAhx-opB-Chol, cholest-5-en-3β-yl-4-(2-oxopropyl)-benzoate; MAChol, 6-cholesteryloxyhexyl methacrylate; MAgala, 6-Omethacryloyl-D-galactopyranose; mPEG, (poly(ethylene glycol) methylether methacrylate; MTC-Chol, cholesteryl 2-(5-methyl-2-oxo-1;3-dioxane-5-carboxyloyloxy)ethyl carbamate); NB, norbornene; OC-ROP, organocatalytic ring-opening polymerization; PAE, poly(β-amino ester); PEG, poly(ethylene glycol); PLL, poly(l-lysine); PTX, paclitaxel; RAFT, reversible addition−fragmentation chain transfer polymerization; ROMP, ring-opening metathesis polymerization; SS, disulfide bridge; TMC, trimethylene carbonate.

**Table 4:** Polymers containing cholesterol moieties in side chains introduced by post-modification.

Polymer	Form of Carrier	Drug or Dye	Mn or Mw (kDa) (Ð)	LE (%)	LC (%)	Dg (%)	Lit.
**Amidation**							
PEI-Chol	liposome	pDNA (pGL3 promoter)	N/A	N/A	N/A	N/A	[134]
HA-Chol	nanogel	rhGH/EPO lysozyme/exendin-4	52.0 (N/A)	N/A	N/A	3.0	[14]
			54.0 (N/A)			7.0	
			59.0 (N/A)			15.0	
			66.0 (N/A)			27.0	
			75.0 (N/A)			42.0	
P(MPC-*co*-NPEM)*-g-*Chol (nMPC:nChol = 86:14)	micelle	DOX	11.2 (1.95)	53.8	21.5	100	[135]
P(MPC-*co*-NPEM)*-g-*Chol*-g-*FA (nMPC:nChol:nFA = 56:14:30)			12.1 (2.16)	53.5	21.4		
P(MPC-*co*-NPEM)*-g-*Chol*-g-*FA (nMPC:nChol = 74:26)	micelle	DOX	6.6 (N/A)	48.6	19.6		[136]
P(MPC-*co*-NPEM)*-g-*Chol*-g-*FA (nMPC:nChol:nFA = 64:27:9)			N/A	52.6	21.1		
P(MPC-*co*-NPEM)*-g-*Chol*-g-*FA (nMPC:nChol:nFA = 61:16:23)		DOX (10% *w*/*w*)		81.9	8.2		
		DOX (20% *w*/*w*)		77.9	15.6		
		DOX (40% *w*/*w*)		62.3	24.9		
		DOX (50% *w*/*w*)		67.4	33.7		
P(MPC-*co*-NPEM)*-g-*Chol*-g-*FA (nMPC:nChol:nFA = 58:11:31)		DOX	11.4 (N/A)	58.8	23.5		
γ-PGA*-g-*Chol	hydrogel	DOX	N/A	N/A	6.39	96.2	[76]
PEI-Chol	polyplex	siRNA	N/A	N/A	N/A	N/A	[137,138]
PEI-Chol (nPEI:nChol = 1:7.5)	micelle	SFB	9.8 (N/A)	N/A	N/A	N/A	[37]
PEI-Chol (nPEI:nChol = 1:15.5)			13.1 (N/A)		13.1		
PEI-Chol-PEG (nPEI:nChol:nPEG = 1:7.5:1)			15.3 (N/A)		N/A		
PEI-Chol-PEG (nPEI:nChol:nPEG = 1:15.5:1)			23.9 (N/A)		N/A		
Chol-CA-Spe	nanogel	siRNA	N/A	N/A	N/A	3.1	[139]
Cyc-PEI-Chol (nChol:nCyc = 0.17)	polyplex	siRNA	28.8 (N/A)	N/A	N/A	N/A	[140]
Cyc-PEI-Chol (nChol:nCyc = 0.33)			32.4 (N/A)				
Cyc-PEI-Chol (nChol:nCyc = 0.53)			36.9 (N/A)				
PEI-Chol	nanoparticle	Ce6	N/A	N/A	35	N/A	[141]
Chol-GC	micelle	DOX	N/A	80.9	10.8	6.1	[142]
Chol-GC-FA				87.0	11.6		
NLS-Chol-GC				77.4	10.4		
NLS-Chol-GC-FA				79.0	10.6		
Chol-GC		Cou6		89.8	1.76		
Chol-GC-FA				87.0	1.71		
NLS-Chol-GC				90.1	1.77		
NLS-Chol-GC-FA				89.6	1.73		
PEI-Chol (nChol:nPEI = 25.8)	polyplex	siRNA	N/A	N/A	N/A	N/A	[143]
PEI-Chol (nChol:nPEI = 52.5)							
PEI-Chol (nChol:nPEI = 102.44)							
F-PEI-Chol (nChol:nF-PEI = 21.3)							
F-PEI-Chol (nChol:nF-PEI = 50.9)							
F-PEI-Chol (nChol:nF-PEI = 105.6)							
PAMD-Chol (17% *w*/*w* of Chol)	polyplex	siRNA	16.7	N/A	N/A	N/A	[144]
PAMD-Chol (25% *w*/*w* of Chol)			18.5				
PAMD-Chol (34% *w*/*w* of Chol)			21.1				
PEI-Chol	N/A	siRNA	N/A	N/A	N/A	N/A	[132]
**Click Reaction**							
PNIPAAm_10_-SS-P(αN_3_CL*-g-*CholPA)_10_	micelle	IMC	6.0 (1.24)	82.8	40.4	N/A	[133]
PNIPAAm_10_-SS-P(αN_3_CL_10_*-g-*PyrePA_3_/-CholPA_7_)			5.7 (1.40)	71.9	35.9		
acL-Chol-PN	nanogel	FITC-BSA	1 020 (N/A)	N/A	N/A	1.7	[145]
acS-Chol-PN			1 130 (N/A)			1.5	
**Hydrazone Formation**							
P(HPMA-co-MA-εAhx-NHNH_2_-co-MA-εAhx-Chol)	nanoparticle	DOX	21.1 (1.65)	98.0	1.7		[19]
		DTXL		95.0	5.5		
P(HPMA-*co*-MA-εAhx-NHNH_2_-*co*-MA-εAhx-opBChol)	micelle	DOX	38.0 (1.8)	N/A	9.4		[20]
P(HPMA-*co*-MA-εAhx-NHNH_2_-*co*-MA-εAhx-Chol_5α_)			24.5 (1.9)		8.1		
P(HPMA-*co*-MA-εAhx-NHNH_2_-*co*-MA-εAhx-Chol_43_)			25.5 (1.8)		8.2		
P(HPMA-*co*-MA-εAhx-NHNH_2_-*co*-MA-εAhx-Chol_5α_)	micelle	DOX	26.6 (1.88)	N/A	8.2	N/A	[21,22]
P(HPMA-*co*-MA-εAhx-NHNH_2_-*co*-MA-εAhx-opBChol)			30.7 (1.65)		11.2		
P(HPMA-*co*-MA-εAhx-NHNH_2_-*co*-MA-εAhx-LevChol)			28.5 (1.89)		10.9		
P(HPMA-*co*-MA-εAhx-NHNH_2_-*co*-MA-εAhx-Chol_43_)			26.8 (1.72)		7.9		
**Nucleophilic Substitution (Br to N)**							
Chol*-g-*P(MSC-PDL)	nanoparticle	plasmid p3XFLAG-CMV-p53	8.3 (1.95)	N/A	N/A	9.7	[129]
		miR-23b					[130]
Chol-PHP	polyplex	pDNA	12.5 (N/A)	N/A	N/A	31.6	[131]
**Supercritical CO_2_-Assisted Spray Drying (SASD)**							
PURE-G_4_-OMeOx_48_[PLGA-Chol]	microparticle	SDF	N/A	N/A	19.1	N/A	[80]
PURE-G_4_-OEtOx_48_[PLGA-Chol]					22.1		
**Boronate Linkage**							
mPEG-PLL*-g-*DHPA/Chol-PBA (DHPA:Chol-PBA = 3:1)	nanoassembly	DOX	N/A	8.1	2.0	N/A	[38]
mPEG-PLL*-g-*DHPA/Chol-PBA (DHPA:Chol-PBA = 3:2)				30.1	7.5		
**Esterification**							
PAE(-SS-mPEG)*-g-*Chol)	nanoparticle	DOX	12.95 (1.45)	55.4	10.8	N/A	[39]
PAE(-SS-mPEG)*-g-*Chol)/PAE-*g*-mPEG-*g*-Chol/(mass ratio = 2:1)	micelle	DOX (10% *w*/*w*)	12.95 (1.45)/8.79 (1.90)	61.2	16.1	N/A	[39,146]
		DOX (20% *w*/*w*)		64.7	26.4		
		DOX (30% *w*/*w*)		55.7	28.8		
PAE(-SS-mPEG)*-g-*Chol)/PAE-*g*-mPEG-*g*-Chol/(mass ratio = 1:1)		DOX (10% *w*/*w*)		63.5	16.7		
		DOX (20% *w*/*w*)		69.8	28.5		
		DOX (30% *w*/*w*)		60.9	31.5		
PAE(-SS-mPEG)*-g-*Chol)/PAE-*g*-mPEG-*g*-Chol/(mass ratio = 1:2)		DOX (10% *w*/*w*)		59.1	15.8		
		DOX (20% *w*/*w*)		63.0	25.7		
		DOX (30% *w*/*w*)		53.9	27.9		
poly(BAC-AMPD)*-g-*PEG*-g-*Chol	micelle	DOX	N/A	27.1	5.4	54.5	[40]
Chol-CS	nanoparticle	ATRA (10% *w*/*w*)	N/A	88.7	8.0	4	[12]
		ATRA (20% *w*/*w*)		82.3	11.8		
		ATRA (40% *w*/*w*)		77.9	24.3		
		ATRA (50% *w*/*w*)		74.0	28.3		
rPAA-Chol	nanoparticle	siRNA	9.7 (N/A)	N/A	N/A	14.0	[147,148]
			10.9 (N/A)			29.0	
			13.5 (N/A)			57.0	
			15.9 (N/A)			87.0	
(PAE*-g-*Chol)*-b-*PEG*-b-*(PAE*-g-*Chol)	micelle	DOX (10% *w*/*w*)	N/A	33.6	4.2	48.0	[41]
		DOX (20% *w*/*w*)		48.7	13.5		
		DOX (50% *w*/*w*)		59.5	20.1		
		DOX (80% *w*/*w*)		55.3	24.3		
PEG-PMMI-CholC6	liposome	RAPA	74.0 (1.51)	76.9	N/A	4.9	[42]
PEG-PMMI-CholC6	liposome	MTX	N/A	63.1	N/A	N/A	[43]
PMMI-CholC6	micelle	PX	57.1 (1.60)	30.0	6.2	4.9	[44]
PEG-PMMI-CholC6			74.3 (1.51)	40.3	8.3	16.4	
Chol-PEG_22_- hbPG_35_	liposome	Atto 488 tetrazine	N/A	>40	N/A	N/A	[45]
		Alexa Fluor 594 azide		>40			
HA-Chol	micelle	α -TOC	N/A	77.6	16.1	4.6	[15]
		CUR		82.8	3.3		
		CoQ10		86.2	10.7		
L-PGA*-g-*Chol	nanoparticle	HSA	N/A	N/A	N/A	0.065	[77]
PEI-CyD*-g-*Chol	micelle	DOX	N/A	N/A	5.4	5.2	[149]
					7.4	7.9	
					12.8	18.6	
Chol-AL-AG	liposome	N/A	27.0 (N/A)	N/A	N/A	N/A	[150]
mPEG-D_labile_-PAE*-g-*Chol	micelle	DOX	N/A	53.5	11.2	55	[46]
Chol-XG	nanogel	PTX	20 000 (N/A)	N/A	N/A	N/A	[151]
Dex-Chol	micelle	RAPA 10%	43.8 (N/A)	79.9	7.3	4	[152]
		RAPA 20%		90.1	12.6		
mPEG*-b-*P(MBC_78_-{*g*-DMDPTA_36_; *g*-Chol_30_}-*co*-LA_110_)	polyplex	miRNA-34a	43.5 (N/A)	N/A	N/A	N/A	[47]
mPEG*-b-*P(MBC_65_-{*g*-DMDPTA_11_; *g*-Chol_19_; *g*-Morph_6_;}-co-LA_120_)			35.4 (N/A)				
Chol-PN	nanoparticle	MTX	N/A	N/A	5.2	3.6	[48]
					6.7	5.7	
					8.6	6.7	
mPEG-PLL*-g-*DHPA/Chol-PBA (DHPA:Chol-PBA 1:1)				55.6	13.9		
Dex-Chol	micelle	DOX	N/A	41.5	6.3	13.0	[153]
HIS-Dex-Chol (24% HIS graft ratio)				46.1	7.6		
HIS-Dex-Chol (46% HIS graft ratio)				56.3	12.3		
Chol*-g-*P(HEMA_10_-*co*-DEAEMA_25_)*-b-*PPEGMA_10_	micelle	DOX (12.5% *w*/*w*)	14.3 (1.47)	20.0	4.1	N/A	[49]
		DOX (25% *w*/*w*)		38.0	8.7		
		DOX (50% *w*/*w*)		30.0	13.1		
Chol*-g-*P(HEMA_10_-*co*-DEAEMA_35_)*-b-*PPEGMA_10_		DOX (12.5% *w*/*w*)	16.4 (1.54)	25.0	4.5		
		DOX (25% *w*/*w*)		48.5	10.8		
		DOX (50% *w*/*w*)		36.7	15.5		
PAE*-g-*mPEG-Chol	micelle	DOX (25% *w*/*w*)	8.8 (1.90)	25.5	9.5	62.0	[50]
		DOX (50% *w*/*w*)		60.0	28.3		
		DOX (100% *w*/*w*)		52.8	30.7		
HMW-Chol*-g-*AlgA	nanoparticle	acetamiprid	112.2 (N/A)	90.8	N/A	4.6	[154]
MMW-Chol*-g-*AlgA			64.5 (N/A)	86.8		5.4	
LMW-Chol*-g-*AlgA			47.5 (N/A)	81.0		5.7	
P(NIPAAm-*co*-NHMAAm)*-g-*Chol	micelle	Py	8.1 (1.40)	N/A	0.4	N/A	[155]
HPC-PEG-Chol-biotin	micelle	PTX	N/A	N/A	8.4	3.6	[51]
CNC-Chol	nanocrystal	FA	N/A	58	N/A	17	[156]
Chol-Imi-OS	nanoparticle	CUR	N/A	17.8	4.2	N/A	[157]

Abbreviations: acL, acid labile; acS, acid stabile; AG, arabinogalactan; AL, alanine; AlgA, alginic acid; AMPD, 4-(aminomethyl)piperidine; ATRA, all-trans retinoic acid; α-azo-caprolactone (αN_3_CL); BAC, *N*,*N*-cystaminebis(acrylamide); CA-Spe, cycloamylose with spermine group; Ce6, chlorin e6; Chol, cholesterol; CholC6, 6-(cholesteryloxycarbonyloxy) hexanol; CholPA, cholestryl 4-pentynoate; CNC, cellulose nanocrystals; coQ10, coenzyme Q10; Cou6, coumarin 6; CS, chitosan; CUR, curcumin; Cyc, cyclam; CyDex, cycloldextrin; DEAEMA, 2-(diethylamino)ethyl methacrylate, Dex, dextrin; Dg, degree of grafting; DHPA, 3-(2;4-dihydroxyphenyl)propionic acid; DMDPTA, *N*,*N*-dimethyldipropylenetriamine; DOX, doxorubicin; EPO, erythropoietin; F, heptafluorobutyric anhydride; FA, folic acid; FITC-BSA, fluorescein isothiocyanate-labeled bovine serum albumin; GC, glycol chitosan; HA, hyaluronic acid; hbPG, hyperbranched poly(glycerol); HEMA, hydroxyethyl methylacrylate; HIS, histidine; HMW, high molecular weight; HPC, hydroxypropyl cellulose; HSA, human serum albumin; IMC, indomethacine; IMI, imidazole; LA, lactic acid; LMW, low molecular weight; L-PGA, poly(l-glutamic acid); miR-23b, micro RNA-23b; MBC, 5-methyl-5-benzylcarboxyl-1,3-dioxan-2-one; MMW, medium molecular weight; morph, 4-(2-aminoethyl) morpholine; MPC, 2-methacryloyloxyethyl phosphorylcholine; mPEG, (poly(ethylene glycol) methylether methacrylate; MSC, *N*-methyldiethanolamine-co-diethyl sebacate; MTX, mitoxantrone; NHMAAm, *N*-hydroxylmethylacrylamide; NIPAAm, *N*-isopropylacrylamide; NLS, nuclear localization signal; NPEM, p-nitrophenyloxycarbonylpoly(ethylene glycol)methacrylate; OS, oxidized-starch; PAE, poly(β-amino ester); PAMD, plerixafor/AMD3100; PBA, poly(3-boronophenyl)carbamate; PDL, ω-pentadecanolide; pDNA, plasmid DNA; PEG, poly(ethylene glycol); PEGMA, poly(ethylene glycol) methyl ether methacrylate; PEI, polyethylenimines; PGA, poly(glutamic acid); PHP, poly[hexamethylene diacrylate-β-(5-amino-1-pentanol)]; γ-PGA, poly(γ-glutamic acid); PLGA, poly(d,l-lactide-co-glycolide); PLL, poly(l-lysine); PMMI, poly(monomethyl itaconate); PN, pullulan; PTX, paclitaxel; PURE-G4-OEtOx48, ethoxylated polyurea; PURE-G4-OMeOx48, methoxylated polyurea; PX, piroxicam; Py, pyrene; PyrePA, pyrenylmethyl 4-pentynoate; RAPA, rapamycin; rPAA, bioreducible poly(amidoamine); SASD, supercritical CO_2_-assisted spray drying; SDF, sildenafil; SFB, sorafenib; SS, disulfide bridge; α-TOC, α-tocopherol;XG, xyloglucan.

## Abbreviations

AAA, ascorbyl acrylate; Achol, cholesteryl acrylate; acL, acid labile; acS, acid stabile; ADR, Adriamycin; AECChol, cholesteryl acryloyoxy ethyl carbonate; AEDA, 2-((2-azidoethyl) disulfanyl) ethan-1-amine hydrochloride; AG, arabinogalactan; AL, alanina; AlgA, alginic acid; AmB, amphotericin B; AMPD, 4-(aminomethyl)piperidine; ATRA, all-trans retinoic acid; ATRP, atom transfer radical polymerization; (αN3CL), α-azo-caprolactone; BAC, *N*,*N*-cystaminebis(acrylamide); BnAAA, benzyl protected ascorbylacrylate; CABA, cabazitaxel; CA-Spe, cycloamylose with spermine group; Ce6, chlorin e6; CF, 5,6-carboxyfluorescein; Chol, cholesterol; CholC6, 6-(cholesteryloxycarbonyloxy) hexanol; CholDEGA, cholesteryl diethyleneglycol acrylate; CholPA, cholestryl 4-pentynoate; CNC, cellulose nanocrystals; coQ10, coenzyme Q10; Cou6, coumarin 6; CPT, S-(+)-camptothecin; CS, chitosan; CUR, curcumin; Cyc, cyclam; CyDex, cycloldextrin; CYS, cystamine; DBU, 1,8-diazabicyklo[5.4.0]undek-7-en, DCC, dicyclohexylcarbodiimide; DEAEMA, 2-(diethylamino)ethyl methacrylate; Dex, dextrin; Dg, degree of grafting; DHPA, 3-(2,4-dihydroxyphenyl)propionic acid; DMA, 1-decyl methacrylate; DMAAm, *N*,*N*-dimethylacrylamide; DMAE, 2-(dimethylamino)ethyl 1H-imidazole-1-carboxylate; DMAEMA, 2-(dimethylamino)ethyl methacrylate; DMAP, 4-dimethylaminopyridine, DMDPTA, *N*,*N*-dimethyldipropylenetriamine; DMEDA, *N*,*N*-dimethylaminoethylamine; DOX, doxorubicin; DP7, antimicrobial peptide (VQWRIRVAVIRK); DTXL, docetaxel; DUP1, peptide (CFRPNRAQDYNTN); DUPA, 2-[3-(1,3- dicarboxypropyl) ureido]pentanedioic acid; EDC, 1-(3-dimethylaminopropyl)-3-ethylcarbodiimide; EPO, erythropoietin; F, heptafluorobutyric anhydride; F68, pluronic F68; FA, folic acid; FITC-BSA, fluorescein isothiocyanate-labeled bovine serum albumin; FITC-CM-Dex, fluorescein isothiocyanate carboxymethyl dextran; Fmoc, 9-fluorenylmethoxycarbonyl; GA, glutamic acid; GC, glycyrrhetinic acid; GCS, glycol chitosan; GEM, gemcitabine; HA, hyaluronic acid; hbPG, hyperbranched poly(glycerol); (HE)_5_, histidine-glutamic acid decapeptide; HEMA, hydroxyethyl methylacrylate; HIS, histidine; HMW, high molecular weight; HPbCD, modified 2-hydroxypropyl-b-cyclodextrin macrocycles; HPC, hydroxypropyl cellulose; HPMA, *N*-(2-hydroxypropyl) methacrylamide; HSA, human serum albumin; Hz, hydrazone; IBU, ibuprofen; ICG, indocyanine green; IMC, indomethacine; IMI, imidazole; LA, lactic acid; LC, lecithin; LMW, low molecular weight; L-PGA, poly(l-glutamic acid); Lys, lysine; MAA, methacrylic acid; MAChol, 6-cholesteryloxyhexyl methacrylate; MA-ɛAhx-Chol, cholest-5-en-3β-yl 6-methacrylamido hexanohydrazide; MA-ɛAhx-Chol43, cholest-4-en-3β-yl 6-methacrylamido hexanohydrazide; MA-ɛAhx-Chol5α, 5α-cholestan-3β-yl 6-methacrylamido hexanohydrazide; MA-ɛAhx-LevChol, cholest-5-en-3β-yl-4-oxopentano 6-methacrylamido hexanohydrazide; MA-ɛAhx-NHNH2, 6-methacrylamido hexanohydrazide; MAgala, 6-Omethacryloyl-D-galactopyranose; MBC, 5-methyl-5-benzylcarboxyl-1,3-dioxan-2-one; MMW, medium molecular weight; morph, 4-(2-aminoethyl) morpholine; MPC, 2-methacryloyloxyethyl phosphorylcholine; mPEG, (poly(ethylene glycol) methylether methacrylate; MSC, *N*-methyldiethanolamine-co-diethyl sebacate; MTC-Chol, cholesteryl 2-(5-methyl-2-oxo-1;3-dioxane-5-carboxyloyloxy)ethyl carbamate); MTX, mitoxantrone; NAS, *N*-acryloxysuccinimide; NB, norbornene; NHMAAm, *N*-hydroxylmethylacrylamide; NIPAAm, *N*-isopropylacrylamide; NLS, nuclear localization signal; NPEM, p-nitrophenyloxycarbonylpoly(ethylene glycol)methacrylate; OC, α-tocopherol; OC-ROP, organocatalytic ring-opening polymerization; opB-Chol, cholest-5-en-3β-yl-4-(2-oxopropyl)-benzoate; OS, oxidized-starch; PAA, poly(acrylic acid); PAE, poly(β-amino ester); PAMAM, polyamidoamine; PAMD, plerixafor/AMD3100; PAMPS, poly(2-(acrylamido)-2-methylpropanesulfonic acid) sodium salt; PAsp(DET), poly{*N*-[*N*-(2-aminoethyl)-2-aminoethyl]aspartamide}; PBA, poly(3-boronophenyl)carbamate; Pbf, 2,2,3,6,7-pentamethyldihydrobenzofuran-5-sulfonyl; PCL, poly(ε-caprolactone); PCLp, polymer-caged lipoplex; PDL, ω-pentadecanolide; PDLA, poly(d-lactide acid); pDNA, plasmid DNA; PEG, poly(ethylene glycol); PEGMA, poly(ethylene glycol) methyl ether methacrylate; PEI, polyethylenimines; PEO, poly(ethylene oxide); PEP, peptide; PF127, Synperonic PE/F 127; PGA, poly(γ-glutamic acid); PHPMAlac, poly(*N*-(2-hydroxypropyl)methacrylamide mono/dilactate); PHP, poly[hexamethylene diacrylate-β-(5-amino-1-pentanol)]; PLA, poly(lactic acid); PLGA, poly(d,l-lactide-co-glycolide); PLGVRK, matrix metalloproteinase-2 responsive peptide; PLL, poly(l-lysine); PLLA, poly(l-lactide acid); PMMI, poly(monomethyl itaconate); PN, pullulan; PplX, protoporphyrin IX; PSO, polyoxyethylene sorbitol oleate; PTX, paclitaxel; PURE-G4-OEtOx48, ethoxylated polyurea; PURE-G4-OMeOx48, methoxylated polyurea; PX, piroxicam; Py, pyrene; PyrePA, pyrenylmethyl 4-pentynoate; QC, quercetin; RAFT, reversible addition−fragmentation chain transfer polymerization; RAPA, rapamycin; RES, resveratrol; (RG)_5_, arginine-glycine decapeptide; RGD, arginylglycylaspartic acid; ROMP, ring-opening metathesis polymerization; ROP, ring-opening polymerization; rPAA, bioreducible poly(amidoamine); SA, succinic anhydride; SASD, supercritical CO_2_-assisted spray drying; SDF, sildenafil; SFB, sorafenib; SS, disulfide bridge; TMC, trimethylene carbonate; TMX, tamoxifen; α-TOC, α-tocopherol; TPGS, tocopheryl poly(ethylene glycol) succinate; TPL, triptolide; TPP, triphenylphosphine; uPA, short peptide sequence for urokinase plasminogen activator; XG, xyloglucan.
